# Enhanced Quick-Cooking Red Beans: An Energy-Efficient Drying Method with Hot Air and Stepwise Microwave Techniques

**DOI:** 10.3390/foods13050763

**Published:** 2024-03-01

**Authors:** Wisanukorn Thonglit, Surachet Suanjan, Prarin Chupawa, Sudathip Inchuen, Wasan Duangkhamchan

**Affiliations:** 1 Research Unit of Process Design and Automation, Faculty of Engineering, Mahasarakham University, Maha Sarakham 44150, Thailand; 2 Research Unit of Mechatronics Engineering, Faculty of Engineering, Mahasarakham University, Maha Sarakham 44150, Thailand; prarin.c@msu.ac.th; 3 Research Unit of Smart Process Design and Automation, Mahasarakham University, Maha Sarakham 44150, Thailand; 4 Department of Food Technology, Faculty of Technology, Mahasarakham University, Maha Sarakham 44150, Thailand; sudathip.i@msu.ac.th

**Keywords:** stepwise drying, quick-cooking grains, response surface methodology, synthetic evaluation index, hybrid drying

## Abstract

This research introduced an energy-efficient drying method combining hot-air drying with stepwise microwave heating for producing quick-cooking red beans. Crucial parameters such as the effective diffusivity coefficient (D_e_), and specific energy consumption (SEC) were examined across varying conditions with the aim of optimizing the drying condition. The results showed that D_e_ and SEC varied in a range of 0.53 × 10^−9^–3.18 × 10^−9^ m^2^·s^−1^ and 16.58–68.06 MJ·(kg·h^−1^)^−1^, respectively. The findings from the response surface methodology indicated that optimal drying conditions for cooked red beans are achieved at a hot air temperature of 90 °C, a microwave power of 450 W (corresponding to an initial intensity of 2.25 W·g^−1^), and a rotational speed of 0.2 Hz. These conditions lead to the maximum effective diffusivity coefficient and the lowest specific energy consumption. Further investigations into step-up (150–300 W to 300–450 W) and step-down (300–450 W to 150–300 W) microwave heating modes were conducted to refine the drying process for enhanced energy efficiency. The synthetic evaluation index revealed that step-down microwave heating strategies of 450 W-to-150 W and 300 W-to-150 W, applied at a temperature of 90 °C and a rotational speed of 0.2 Hz, were notably effective. These methods successfully minimized energy use while preserving the quality attributes of the final product, which were comparable to those of traditionally cooked and freeze-dried red beans. The combined approach of hot-air drying with step-down microwave heating presents a promising, energy-saving technique for producing quick-cooking beans that retain their rehydration qualities and texture.

## 1. Introduction

Renowned for their rich nutritional components such as proteins, fiber, and beneficial phytochemicals [1,2,3], red beans hold a valuable place in our diets. However, conventional preparation techniques requiring prolonged soaking and cooking times [4] limit their daily consumption. While certain approaches strive to reduce these durations, like chemical treatments or pressure cooking, they often compromise the nutritional profile and sensory attributes of the beans. Hot-air drying and microwave heating techniques, deployed independently, have been recognized for their efficiency and quality retention across various food processing applications [5].

The exploration of drying technologies for legumes, particularly red beans, is a pivotal area in advancing both culinary efficiency and the preservation of nutritional value. Traditional methods, such as hot-air drying and the integration of constant microwave power, have paved the way for significant improvements, yet they also bring to light several critical limitations. Among these, the risk of overheating or charring during the final drying stages due to uneven microwave intensity stands out, further complicated by the operational challenges and inefficiencies associated with adjusting microwave power dynamically. This situation is exacerbated by the need for precise control over the drying process to avoid quality degradation [6,7]. Additionally, the advent of microwave radiation technology has revolutionized the drying landscape by significantly reducing drying times and improving the texture and quality of cooked beans, marking a significant step forward in drying technology and offering a promising avenue for future research and application [7]. This enhanced understanding underscores the potential of microwave-assisted drying methods in overcoming traditional barriers, highlighting the need for ongoing innovation in drying techniques to achieve optimal drying efficiency without compromising bean quality.

Achieving a balanced approach in drying technologies, especially regarding energy efficiency and quality preservation under variable microwave power, presents a significant challenge. This complexity is further explored in studies focusing on microwave hot-air rolling drying (MHRD) and its impact on kidney beans, where the reduction in drying times is weighed against the necessity for maintaining quality, necessitating precise adjustments in drying parameters [8,9]. Additionally, research into pre-treatment methods and the synergetic effects of combined drying techniques offers insights into quality enhancement for beans. These studies pinpoint a critical gap in current methodologies: the absence of an optimized technique that adeptly balances drying efficiency with quality preservation amid fluctuating conditions [7]. This highlights the need for approaches that can navigate the intricacies of drying parameters to achieve optimal outcomes.

The emphasis on storage stability and the impact of various drying methods on bean structural integrity underscores the need for precision in the drying process. Studies have explored the role of innovative pre-treatments and drying combinations in enhancing both drying efficiency and product quality, suggesting that new approaches could significantly improve outcomes [4,10,11,12,13]. Additionally, addressing challenges like nonuniform heating through advanced drying techniques represents a frontier in drying technology, aiming to achieve uniform quality and efficiency [14,15,16,17,18]. However, finding a solution that integrates the ease of practical application with the simplicity of operation continues to be a challenge, highlighting the need for further investigation in this area.

The objectives of this study were twofold: to propose an energy-efficient drying method for quick-cooking red beans using a combined approach of hot-air drying and microwave heating, and to explore the influence of key drying parameters on drying performance under varied conditions. Our investigation was comprehensive, taking into account the different aspects that affect the drying process and its outcomes. We sought to identify the optimal drying conditions that would result in minimal energy consumption and maximum drying efficiency. Furthermore, this study assessed the potential of stepwise microwave heating to prevent the degradation of the final product quality. We aimed to identify the most effective drying process that would ensure both energy efficiency and optimal product quality.

## 2. Materials and Methods

### 2.1. Materials and Sample Preparation

Adzuki red beans were acquired from a supermarket in Maha Sarakham Province, Thailand. These beans were carefully selected for their oval shape and size, measuring 5–9 mm in length, 4–6 mm in width, and 4–6 mm in thickness, with each grain weighing between 50 and 250 mg. Following selection, the beans were rinsed and then submerged in warm distilled water (~60 °C) at a grain-to-water ratio of 1:3. This soaking process took place for 2 h in a thermally insulated container. After soaking, 150 g of the grains were cooked in 450 mL of boiled water for 45 min. During the cooking process, the sample was checked by compressing one kernel between two glasses until the uncooked chalky core disappeared [19]. Finally, the completely cooked grains were packed in an aluminum foil bag before being subjected to the drying process. Samples prepared by soaking in excess water for 7 h and cooking for 45 min in boiling water were considered as traditionally cooked red beans, following [20] with some modifications.

### 2.2. Experimental Setup

**Figure 1:**
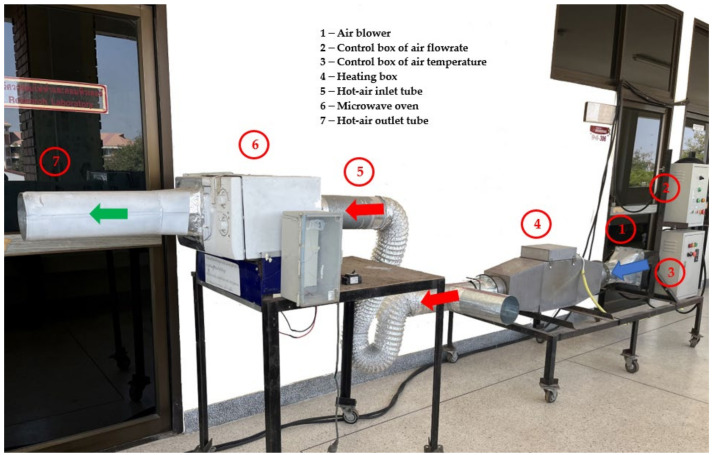
Lab-scale combined hot-air/microwave dryer: Blue arrow represents flow direction of inlet air; Red arrow represents flow direction of hot air and green arrow represent the outlet air flow.

A domestic microwave oven (2.450 GHz., MS23F300EEK, Samsung, Chon Buri, Thailand) was modified so that hot air could be introduced into an oven cavity, as shown in Figure 1. The direction of air flow is indicated by arrows. The ambient air is denoted by a blue arrow, the hot air by a red arrow, and the moist warm air by a green arrow. Ambient air, supplied by a blower (number 1), was heated in a 10-kW electric box (number 4) before entering the microwave oven (number 6) through an inlet tube (number 5). Hot air with flow rate of 0.02 m^3^·s^−1^ was introduced into the drying chamber with temperature controlled using a PID temperature controller (Model MAC-3D, Shimax Co., Ltd., Akita, Japan). Rotational speed and microwave power were controlled using a microcontroller. For each experimental run, 200 g of cooked samples was placed on a drying disc. The three operating parameters tested were a hot air temperature ranging from 70 °C to 90 °C, a microwave power of 150–450 W, corresponding to initial intensities ranging from 0.75 to 2.25 W·g^−1^, and a rotational speed from 0.07 to 0.20 Hz. A 3^3^ full factorial design was used to study the effects of these parameters to optimize the drying conditions. As they were pre-cooked prior to drying, the dried samples were named ‘quick-cooking red beans’ (QCRB) and are referred to in this way throughout this paper.

### 2.3. Page Drying Modeling

Different drying conditions were tested to study the effects of hot-air drying combined with microwave heating on the cooked red beans. The samples were weighed every 5 min using a 3-digit balance to determine changes in moisture content over time. Experimental runs were stopped when the moisture content reached a steady state or approximately 10% (wet basis, wb). Thin-layer drying modeling was conducted using a simplified Equation (1) to describe the drying behavior, with moisture ratio (MR) expressed as a function of drying time (*t*, min). The measured MR was fitted to the semi-empirical Page models expressed in Equation (2), obtained from different operating conditions.

(1) $$\mathrm{MR}=\frac{M_{t}}{M_{0}}$$

(2) $$MR=\exp(-kt^{n})$$

In Equation (1), M denotes moisture content dry basis (% db), and subscript 0 and *t* represent moisture content at initial drying stage and at any time, respectively. The k and n constants in Equation (2) represent a drying rate constant and a power constant, respectively, estimated by using a nonlinear regression.

The model constants (k and n) obtained from the Page drying equation were then correlated with hot air temperature (T), microwave power (MW), and rotational disc speed (RDS), using a quadratic relationship, expressed by:

(3) $$\mathrm{Constant}=a_{0}+a_{1}T+a_{2}MW+a_{3}RDS+a_{4}(T)(MW)+a_{5}(T)(RDS)+a_{6}\left(MW\right)\left(RDS\right)\;\;\;+a_{7}{\left(T\right)}^{2}+a_{8}{\left(MW\right)}^{2}+a_{9}{\left(RDS\right)}^{2}\;$$

The Page drying model incorporated with a quadratic correlation obtained from Equation (3) was further used to evaluate drying efficiency and optimization aspects.

### 2.4. Effective Diffusivity Coefficient Analysis

In the exploration of drying processes, the theory of diffusion plays a central role, particularly during the falling-rate period where internal moisture movement is crucial. This period is characterized by the diffusion of moisture from within biological materials, dictating the drying dynamics. The effective diffusivity coefficient (D_e_) was estimated using a semi-theoretical equation, which is a simplification from Fick’s second law of diffusion for spherical bodies (as presented in Equation (4)) [11]. For spherical materials with radius r, Equation (4) was converted into a natural logarithm, thereby allowing the establishment of a linear relationship between ln(MR) and drying time (*t*). The slope of this straight line was determined and considered to be the D_e_ value.

(4) $$MR=\frac{6}{\pi^{2}}\exp\left(-\frac{\pi^{2}}{r^{2}}D_{e}t\right)$$

### 2.5. Specific Moisture Evaporation Rate and Specific Energy Consumption

In this work, the specific moisture evaporation rate (SMER) and the specific energy consumption (SEC) used by Mokhtarian et al. [21] were slightly adapted for evaluating the drying system’s efficiency. The alternative definition provides insights into how effectively energy is used in real-time during the drying process, emphasizing the dynamic relationship between energy input and moisture removal rate.

SMER is defined as the rate of water evaporated per unit of energy consumed. It quantifies the efficiency of the drying process by measuring how effectively energy is utilized to remove moisture. SMER was calculated by

(5) $$SMER=\frac{W_{evap}}{E}$$

where W_evap_ represents the moisture evaporation rate (kg·h^−1^) and E the total energy consumed by the hot-air supply system and microwave oven measured in kWh.

SEC represents the amount of energy required to evaporate a specific rate of water during the drying process. It indicates the energy efficiency, focusing on the relationship between energy input and the moisture removal rate over time. By converting energy consumed from kWh to MJ using a factor of 3.6 (MJ·kWh^−1^), SEC was therefore calculated using Equation (6) and expressed in MJ·(kg·h^−1^)^−1^, reflecting the energy efficiency in terms of moisture evaporation rate.

(6) $$SEC=\frac{3.6}{SMER}$$

### 2.6. Stepwise Drying Procedure

The semi-empirical Page drying model incorporated with a quadratic correlation with all drying factors, including hot air temperature, microwave power, and rotational disc speed, was used to estimate the drying time for each stage. The drying period was divided into two steps using 70% of the initial moisture content [22] as an intermediate point for changing microwave intensity. After reaching such a point, with simultaneous drying using both microwave heating and hot air at 90 °C and a rotational disc speed of 0.20 Hz, the cooked sample was further dried until its moisture content reduced to a desired level of 10% wb. Two drying modes including step-up and step-down microwave heating were studied, as shown in Table 1.

### 2.7. Analysis of Quality Attributes

#### 2.7.1. Moisture Content and Water Activity

The standard oven method (AOAC) was used to measure the moisture content of the red beans. Briefly, 3 g of a sample was dried in a hot-air oven at 105 °C for 72 h. Water activity (a_w_), a crucial parameter indicating the amount of water supporting microorganism growth, was measured using a water activity meter (Aqualab, Decagon, WA, USA).

#### 2.7.2. Color Change

The color change of the dried red beans against unprocessed grains was evaluated using the CIE (L* a* b*) color system, where L*, a*, and b*, represent lightness, redness, and yellowness, respectively, and measured using a Minolta Colorimeter (Konica Minolta Sensing Americas, New York, NJ, USA). The total color change (ΔE), expressed in Equation (7), was determined by calculating the difference in lightness (ΔL*), redness (Δa*), and yellowness (Δb*) between the dried and raw beans. This approach provided a detailed assessment of the color impact of the drying process on the beans.

(7) $$\Delta E=\sqrt{{\left(\Delta L*\right)}^{2}+{\left(\Delta a*\right)}^{2}+{\left(\Delta b*\right)}^{2}}$$

#### 2.7.3. Rehydration Properties

Rehydration properties in terms of rehydration ratio, rehydration rate constant, and rehydration time were measured to represent the capability of water absorption during the rehydration process of the dried red beans [4,10,23].

The rehydration ratio (RR) was evaluated by rehydrating 10 g of dried red beans in 100 mL of boiled water for 10 min. The RR value was expressed as the ratio between the rehydrated sample weight (w_r_) and the initial sample weight (w_i_) using Equation (8).

(8) $$RR=\frac{w_{r}}{w_{i}}$$

The rehydration rate constant (k_r_) measures the rate at which water is absorbed during the rehydration process. To determine this rate, 15 g of dried red beans was placed in 1 L of boiling water. The weight of the sample was recorded every minute (w_t_) until it reached equilibrium (w_e_). The weight gain on rehydration (WGR) expressed as Equation (9) was fitted to the semi-empirical first-order kinetic model (Equation (10)), and the rehydration rate constant was then estimated using a nonlinear regression method.

(9) $$WGR=\frac{w_{e}-w_{t}}{w_{e}}\times 100$$

(10) $$WGR=WGR_{e}-\left(WGR_{e}-1\right)\exp\left(-k_{r}t\right)$$

The rehydration time (RT) was measured according to Luithui and Meera [19] with some modifications. Briefly, dried red beans (10 g) were immersed in 1 L of boiling water. A single grain kernel was removed at time intervals of 5 min and later 30 s, and pressed between two glass slides. RT was evaluated at the time at which the grain had no uncooked chalky core.

#### 2.7.4. %Shrinkage and %Breakage

During the drying process, the reduction in solid volume frequently arises from water evaporation. The initial volume (v_i_) and final volume (v_f_) were determined utilizing the liquid displacement method based on the Archimedes principle. This method involved measuring the initial and final volume of the beans by submerging a sample in n-heptane within a 500 mL volumetric flask at room temperature. The volume change, indicative of shrinkage due to water evaporation, was calculated from the known weight and volume of the n-heptane, excluding air space between grains. The shrinkage percentage was derived by examining the change in volume relative to the initial volume, as expressed in Equation (11).

(11) $$\%\;Shrinkage=\frac{v_{i}-v_{f}}{v_{i}}\times 100$$

Microwave drying is effective for its energy efficiency and optimized drying outcomes. However, as the drying process progresses and the sample weight decreases due to moisture loss, the relative intensity of microwave energy per unit of remaining sample weight increases. This phenomenon, while beneficial for drying efficiency, can increase the risk of kernel damage due to the puffing effect. To mitigate this, our study closely examined physical characteristics, especially breakage percentage, using Equation (12).

(12) $$\%\;Breakage=\frac{Total\;\;weight\;\;of\;\;broken\;\;\mathrm{kernels}}{Total\;\;weight}\times 100$$

#### 2.7.5. Textural Properties

A texture analyzer (Stable Microsystems, TA-XT2i, Surrey, UK) was used to determine the hardness and stickiness of the rehydrated red beans following the method of Luithui and Meera [19]. Ten kernels of cooked or rehydrated red beans were placed on a platform and compressed to 80% strain using a 35 mm cylindrical plunger at pre-test and test speeds of 1 mm·s^−1^. The hardness and stickiness values were obtained from the compressive force vs. the distance curve and expressed as maximum force and negative force area, respectively. Stickiness, initially derived from the negative force-time area, was reported as positive values, simplifying interpretation and aligning with conventional presentation standards. The results were compared to traditionally cooked samples and rehydrated samples prepared by freeze-drying.

To prepare the freeze-dried sample, the cooked red beans were uniformly spread on trays, maintaining a thickness of approximately 2 cm. These trays were subsequently placed in a blast freezer set at −20 °C and were allowed to remain there overnight. The following day, the trays were shifted to a freeze dryer (Heto Power Dry PL3000, Thermo Fisher Scientific Inc., Waltham, MA, USA) at 0.5 mbar −40 °C for 24 h. After drying, the freeze-dried red beans were kept under vacuum storage pending further use or analysis.

### 2.8. Response Surface Methodology and Process Optimization

Response surface methodology (RSM), as a powerful tool for defining the correlation between input and output variables [24], was used to investigate the effects of drying parameters consisting of hot air temperature (T), microwave power (MW), and rotational disc speed (RDS) on the effective diffusivity coefficient and energy consumption. RSM associated with a nonlinear polynomial equation (Equation (13)) was then used to optimize a dependent parameter. Three operating factors, (X_1_, X_2_, and X_3_), with three levels for each factor, were quadratically correlated with two responses (Y_1_, and Y_2_), associated with a full factorial experimental design as Equation (13). X_1_, X_2_, and X_3_ were defined as the control factors of drying temperature (°C), microwave power (W), and rotational disc speed (Hz), respectively, while Y_1_ and Y_2_ were the responses of the effective diffusivity coefficient (m^2^·s^−1^) and specific energy consumption (MJ·(kg·h^−1^)^−1^), respectively.

(13) $$Y_{i}=a_{0}+a_{1}X_{1}+a_{2}X_{2}+a_{3}X_{3}+a_{4}X_{1}\cdot X_{2}+a_{5}X_{1}\cdot X_{3}+a_{6}X_{2}\cdot X_{3}+a_{7}X_{1}^{2}+a_{8}X_{2}^{2}+a_{9}X_{3}^{2}$$

The desirability function, d_i_(Y_i_), was utilized to ascertain optimal drying conditions, based on the criteria of maximized D_e_ and minimized SEC, using Equations (14) and (15), respectively [25].

(14) $$d_{i}(y_{i}(x))=\left\{\begin{array}{ll} 0 & if\;y_{i}(x)\;<\;L_{i}\\ {\left(\frac{y_{i}(x)-L_{i}}{U_{i}-L_{i}}\right)}^{s} & if\;L_{i}\leq \;y_{i}(x)\;\leq \;U_{i}\\ 1 & if\;y_{i}(x)\;>\;U_{i} \end{array}\right.$$

(15) $$d_{i}(y_{i}(x))=\left\{\begin{array}{ll} 1 & if\;y_{i}(x)\;<\;L_{i}\\ {\left(\frac{U_{i}-y_{i}(x)}{U_{i}-L_{i}}\right)}^{t} & if\;L_{i}\leq \;y_{i}(x)\;\leq \;U_{i}\\ 0 & if\;y_{i}(x)\;>\;U_{i} \end{array}\right.$$

where, L_i_ and U_i_ are the lower and upper acceptable values for the response, respectively. s and *t* are a weight determining the importance of the response being near its maximum and minimum value, respectively. This mathematical approach optimizes multiple process responses simultaneously, assigning each one value between 0 and 1, where 1 signifies the utmost desirability and 0 denotes unsatisfactory outcomes. The desirability functions for each response for maximum and minimum value were defined and subsequently used maximizing the global desirability (D) expressed in Equation (16) with m representing the total number of responses [25]. The attainment of the highest global desirability (D) indicated the compromise solution, signifying the most effective set of conditions determined for the drying.

(16) $$D={\left(d_{1}\cdot d_{2}\cdot d_{3}\cdot \ldots \cdot d_{m}\right)}^{1/m}$$

In order to evaluate each of the drying methods investigated by a single process indicator, a synthetic evaluation index (S) was constructed [26], considering the relative SEC value (Z_1_), rehydration time (Z_2_), rehydration ratio (Z_3_), %Break (Z_4_), %Shrink (Z_5_), ΔE (Z_6_), and hardness (Z_7_). The quality parameters measured in this study were ranked in order of decreasing significance (presumed from an energy consumption point of view) as follows: SEC, RT, RR, %Breakage, %Shrinkage, ΔE, and hardness, and they were assigned weights (λ_1_, λ_2_, λ_3_, λ_4_, λ_5_, λ_6_, and λ_7_) of 0.7, 0.6, 0.5, 0.4, 0.3, 0.2, and 0.1, respectively. The synthetic evaluation index [27] was then calculated for each of the experimental conditions from Equation (17):

(17) $$S=\sum_{i=1}^{7}\lambda_{i}Z_{i}$$

where $Z_{1}=1-\frac{SEC-SEC_{\min}}{SEC_{\max}-SEC_{\min}}$, $Z_{2}=1-\frac{RT-RT_{\min}}{RT_{\max}-RT_{\min}}$, $Z_{3}=\frac{RR-RR_{\min}}{RR_{\max}-RR_{\min}}$, $Z_{4}=1-\frac{\%Break-\%Break_{\min}}{\%Break_{\max}-\%Break_{\min}}$, $Z_{5}=1-\frac{\%Shrink-\%Shrink_{\min}}{\%Shrink_{\max}-\%Shrink_{\min}}$, $Z_{6}=1-\frac{\Delta E-\Delta E_{\min}}{\Delta E_{\max}-\Delta E_{\min}}$, $Z_{7}=1-\frac{Hardness-Hardness_{\min}}{Hardness_{\max}-Hardness_{\min}}$.

### 2.9. Statistical Analysis

One-way analysis of variance (ANOVA) was utilized, supplemented by Duncan’s multiple range test at a 95% confidence interval, for the comparison of values. Within the framework of RSM, the significance of the model was determined using ANOVA with a threshold of *p* < 0.05. Variables were evaluated for their impact on the response variable, with significance indicated by the lowest *p*-values or highest F-ratios. The model efficacy was assessed through the evaluation of significant terms, regression equation *p*-values, lack-of-fit test *p*-values, and coefficients of determination (R^2^). Additionally, the model adequacy in fitting the experimental data was reflected by high-adjusted and predicted R^2^ values.

## 3. Results and Discussion

### 3.1. Page Drying Model

In our study, the Page model, widely recognized for its applicability in elucidating the drying characteristics of biological materials [28,29], was employed to fit the moisture ratio (MR) observed under various drying conditions. While acknowledging the critique of the Page model’s simplicity and its parameters’ limited physical interpretability, we emphasize its utility in providing an initial understanding of drying kinetics. The parameters k and n, derived from experimental data, were correlated with air temperature (T; °C), microwave power (MW; W), and rotational disc speed (RDS; Hz) through a quadratic relationship, enriching our analysis. This approach not only aligns with established practices but also serves as a foundation for further, more complex modeling efforts, aiming to enhance the practical applicability of our findings in drying technology. Therefore, the complete Page drying equation (Equation (2)) for describing the behavior of drying cooked red beans with convective hot-air drying combined with microwave heating can be used with the correlations of the k and n parameters, as expressed in Equations (18) and (19), respectively.

k = −0.014979 + 0.000297 (T) + 0.00003 (MW) + 0.038093 (RDS) − 0.001208 (T)(RDS) − 0.000028 (MW)(RDS) − 0.000001 (T)^2^ + 0.215454 (RDS)^2^ (18)

n = 2.292883 − 0.027368 (T) + 0.001602 (MW) − 3.67625 (RDS) − 0.000022 (T)(MW) + 0.092591 (T)(RDS) + 0.002012 (MW)(RDS) + 0.000168 (T)^2^ + 0.000001 (MW)^2^ − 13.7099 (RDS)^2^ (19)

### 3.2. Influences of Drying Factors on Drying Efficiency and Energy Consumption

Response surface methodology was conducted with three operating factors including hot air temperature (X_1_), microwave power (X_2_), and rotational disc speed (X_3_), with responses consisting of the effective diffusivity coefficient (D_e_), and specific energy consumption (SEC). All factors and response values presented in Table 2 were fitted to a quadratic equation and optimized using analysis of variance (ANOVA), as shown in Table 3.

The analysis of variance analysis results of drying factors focusing on the quadratic relationship of all responses are presented in Table 3. In Table 3, the effective diffusion coefficient (D_e_) shows that the quadratic model was significant with a *p*-value of less than 0.0001 and an F-value of 13.37. The monomial terms of T and MW were significant at *p* < 0.05 and *p* < 0.001, respectively, while RDS was not significant. Interaction and quadratic terms, having *p*-values exceeding 0.05, were found to be statistically insignificant. This led to the conclusion that a simple linear model adequately represents the data. The “Pred R^2^” value of 0.6656 was slightly different from the “Adj R^2^” value of 0.8107. The “Adeq Precision” value was 11.41 and higher than the desired value of 4, indicating an adequate signal. Therefore, the model was suitable for navigating the design space. The analysis of the drying process of red beans using hot air combined with microwave heating, as indicated by the F-value in Table 3, reveals a clear hierarchy of influencing factors. Microwave power, with an F-value of 110.05, emerges as the most significant factor, followed by hot air temperature (F-value 4.96) and rotational disc speed (F-value 2.70). This finding is comprehensively supported by the reasons outlined in [7]. The significance of microwave energy lies in its direct impact on water molecules, greatly improving the process of moisture movement and evaporation. This interaction leads to a rapid increase in effective moisture diffusivity, a crucial indicator of drying efficiency. Additionally, microwave power uniquely alters the crystalline structure and gelatinization of bean starch, contributing significantly to the overall drying process. These effects, combined, position microwave power as a pivotal factor in the drying efficiency of red beans, far exceeding the influence of hot air temperature and rotational drum speed. 

**Figure 2:**
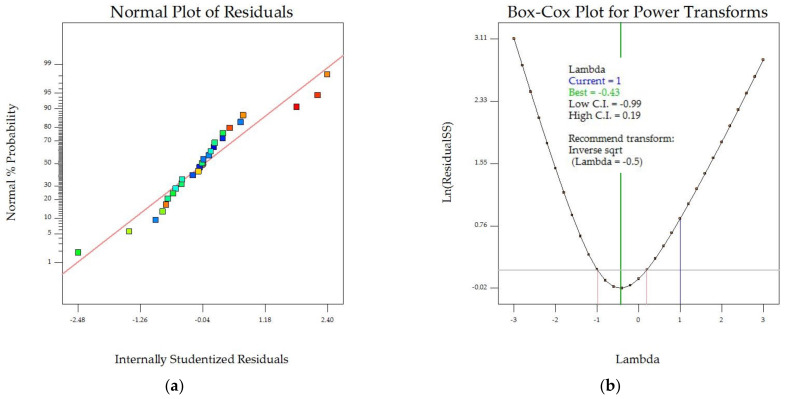

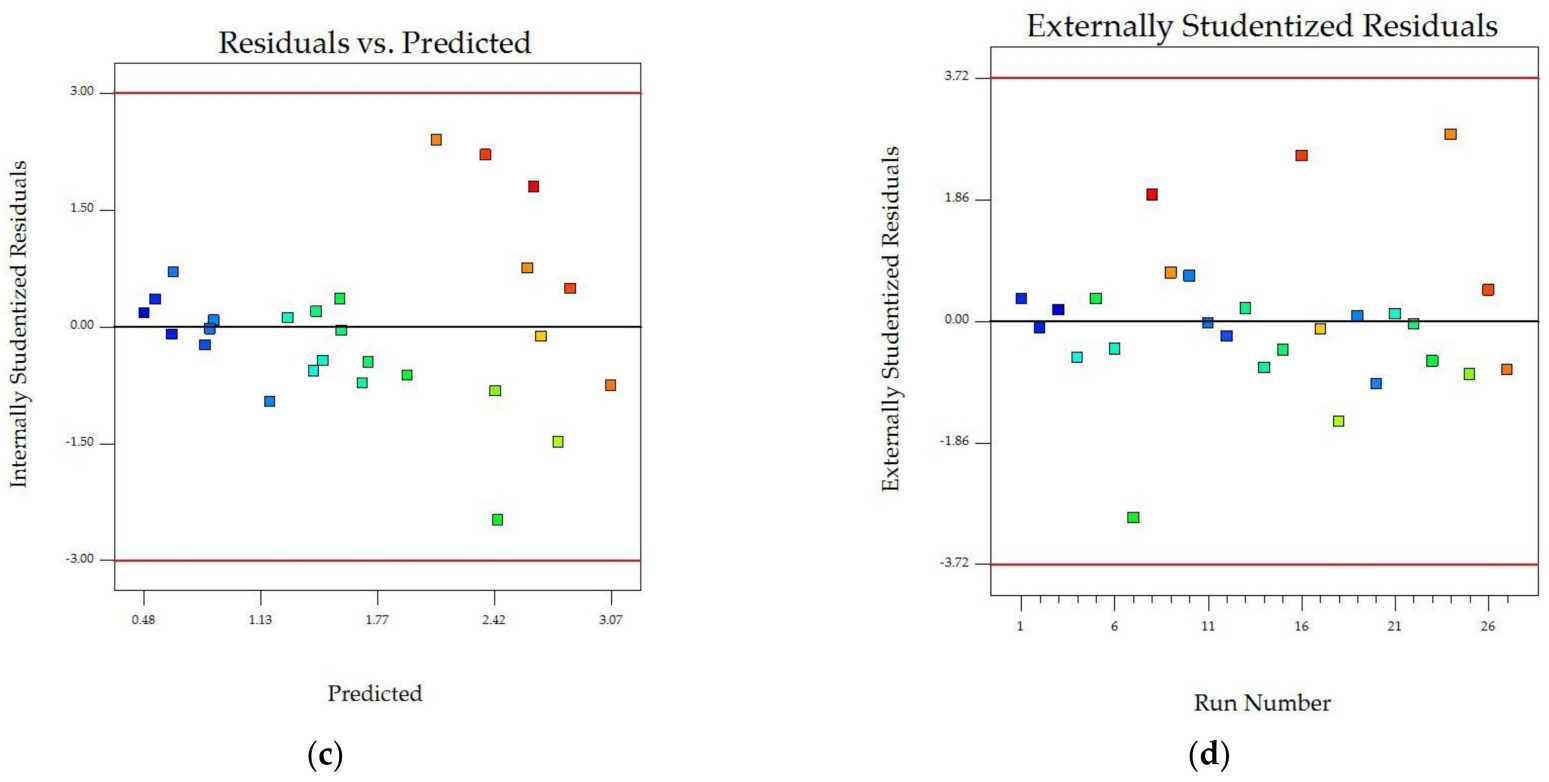
Model diagnostic and influence plots: (**a**) normal probability vs. studentized residuals, (**b**) Box–Cox for power transformation, (**c**) residuals against predicted effective diffusivity coefficient, and (**d**) residuals against run number. Color points by value of D_e_, ranging from minimum value of 0.53 × 10^−9^ m^2^⋅s^−1^ (blue) to maximum value of 3.18 × 10^−9^ m^2^⋅s^−1^ (red).

Figure 2 summarizes the model diagnostic and influence plots of the effective diffusivity coefficient (D_e_). The normal plot of residuals in Figure 2a shows that the model accurately explained the relationship between drying factors and D_e_ value. However, the Box–Cox transformation, which uses a power function to adjust experimental data for statistical analysis [30], shows that the current lambda (1) was not in the confidence interval of 95% (−0.99 ≤ λ ≤ 0.19). In this case, an inverse square transformation (lambda = −0.5) was recommended. Figure 2c shows that the residuals were randomly distributed between −3 and +3. A plot of externally studentized residuals for each experiment run number for the D_e_ value (Figure 2d) revealed that most produced low residuals without any outlier.

**Figure 3:**
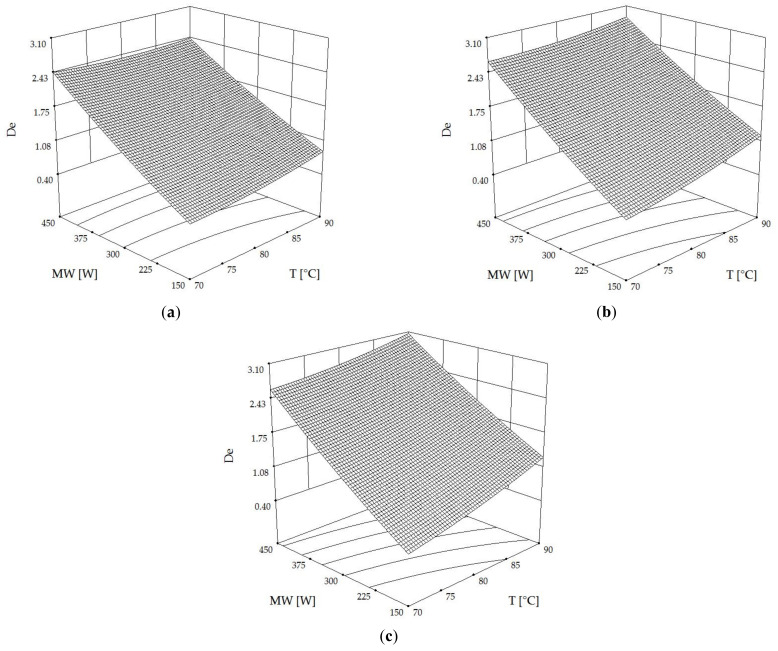
Three-dimensional contour plots of the effective diffusivity coefficient (D_e_, ×10^−9^ m^2^·s^−1^) as affected by hot air temperature (T) and microwave power (MW) at rotational speeds (RDS) of (**a**) 0.07 Hz, (**b**) 0.13 Hz and (**c**) 0.20 Hz.

The effective diffusivity coefficient (D_e_) measures how quickly water or moisture inside a material can diffuse to its surface during the falling-rate period of drying. A higher D_e_ value means faster drying and a shorter drying time [31,32]. According to Table 2, the D_e_ values, ranging from 0.53 to 3.18 × 10^−9^ m^2^·s^−1^, deviate from the standard range of 10^−11^–10^−10^ m^2^·s^−1^ observed in grains subjected to conventional hot-air drying [33]. This discrepancy is attributed to our approach of integrating microwave heating with hot-air drying methods, enhancing the volumetric heating and, consequently, the rate of moisture diffusion. Such outcomes find support in comparable research, specifically the study by Li et al. [11], which documented D_e_ values for pregelatinized kidney beans dried through a hybrid method of hot-air and microwave drying, falling within a similar range. Figure 3a–c display 3D contour plots of D_e_ values based on hot air temperature (T) and microwave power (MW) at different rotational speeds. The plots revealed that drying temperature and microwave power positively influenced D_e_ values at all speeds, with linear relationships. At 0.20 Hz (Figure 3c), the highest D_e_ value (3.1 × 10^−9^ m^2^·s^−1^) was achieved by drying at 90 °C with 450 W microwave heating, whereas the lowest D_e_ value (~0.4 × 10^−9^ m^2^·s^−1^) was obtained at the lowest temperature and microwave power level at all speeds. This observation aligns with insights from the study of Li et al. [7], which emphasizes the significant role of microwave power in influencing the drying efficiency, moisture migration, gelatinization degree, and texture profile of kidney beans. Similarly, Li et al. [11] and Wiset et al. [9] provide evidence of microwave power influence on moisture diffusivity and drying efficiency, further supporting these findings. Specifically, the increase in effective moisture diffusivity (D_e_) with higher microwave power, as observed in [12], is a testament to the microwave capability to enhance molecular movement and water vapor movement within the product. Moreover, the observation in [34] that higher drying temperatures require higher microwave power, leading to more intensive moisture evaporation, corroborates the linear relationship between microwave intensity and D_e_ values. However, it is important to note that while microwave power significantly impacts drying efficiency, as discussed in [8], there is a threshold beyond which further increase may lead to the degradation of bioactive compounds, underscoring the need for optimal microwave power settings in drying processes. When increasing rotation speed, the D_e_ value tended to increase due to more uniform electromagnetic energy absorption. The increase in the effective diffusivity coefficient (D_e_) with rising rotation speeds can be theoretically underpinned by the mechanism of enhanced uniformity in electromagnetic energy absorption. This concept is supported by findings in various studies. For instance, Li et al. [7] and Li et al. [11] demonstrate that the movement or rolling of samples in microwave hot-air drying systems facilitates even exposure to drying agents, leading to more efficient moisture removal. Similarly, Kaveh et al. [12] establishes that higher rotation speeds in microwave-rotary drying significantly improve moisture diffusivity and drying efficiency, likely due to the more consistent energy distribution and absorption by the material. These insights collectively suggest that the increased D_e_ values observed at higher rotation speeds in the present study are a result of more uniform electromagnetic energy absorption across the material, optimizing the drying process.

In addition to the effective diffusivity coefficient (D_e_), in this work, the specific energy consumption (SEC) was evaluated to analyze drying effectiveness. Table 3 displays the significant second-order polynomial function model of SEC, with a *p*-value of 0.0001 (*p* < 0.05) and an F-value of 12.16 (>4). Monomial factors of T and RDS were insignificant model terms, while the MW factor and their interaction term of T*RDS were significant, with an extremely low *p*-value less than 0.001 and 0.005, respectively. The “Pred R^2^” value of 0.6004 was in reasonable agreement with the “Adj R^2^” value of 0.7984. The signal-to-noise ratio was measured using the “Adeq Precision” value, with a desirable value being greater than 4. The “Adeq Precision” was 11.12, indicating an adequate signal. The model was therefore used for further optimization. Among the three factors tested, MW was the most important one as it had the highest F-value, while hot air temperature was the least pronounced parameter.

**Figure 4:**
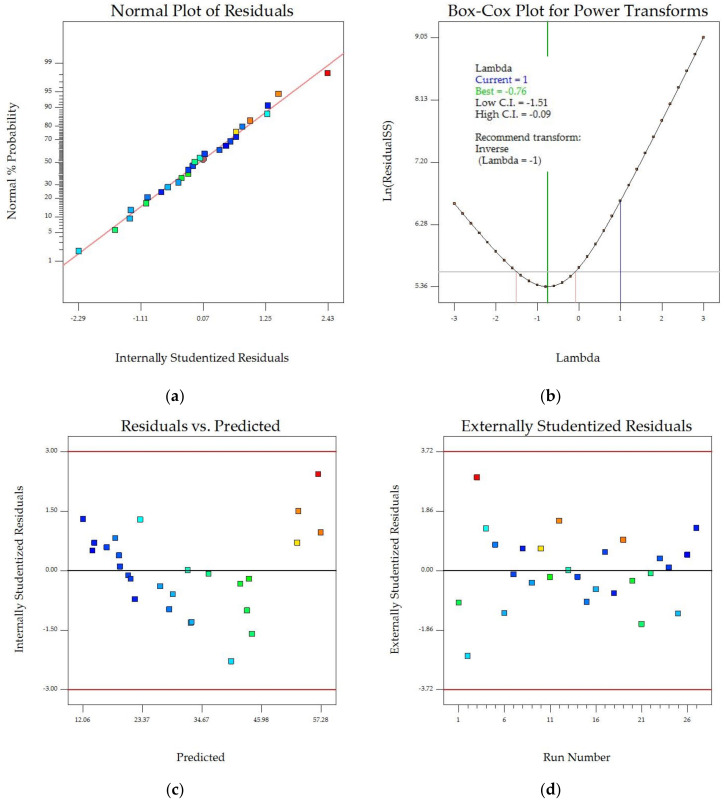
Model diagnostic plots: (**a**) normal probability vs. studentized residuals, (**b**) Box–Cox for power transformation, (**c**) residuals against predicted SEC, and (**d**) residuals against run number. Color points by value of SEC, ranging from minimum value of 16.58 MJ·(kg·h^−1^)^−1^ (blue) to maximum value of 68.06 MJ·(kg·h^−1^)^−1^ (red).

Diagnostic plots were used to ensure the accuracy of our SEC model, as shown in Figure 4. These plots examined the normality of residuals, constant error, outliers, and power transformation. The normal probability plot in Figure 4a shows a strong correlation between observed and predicted data that closely aligned along a diagonal line, but the Box–Cox plot (Figure 4b) illustrates a necessity of function transformation as the current lambda of −1 was inside the confidence interval of 95% (−1.51 ≤ λ ≤ −0.09). Figure 4c shows that the residuals were randomly distributed between −3 and +3. No outlier was observed in the externally studentized residual plot (Figure 4d) suggesting that the model was adequate for predicting experimental data.

**Figure 5:**
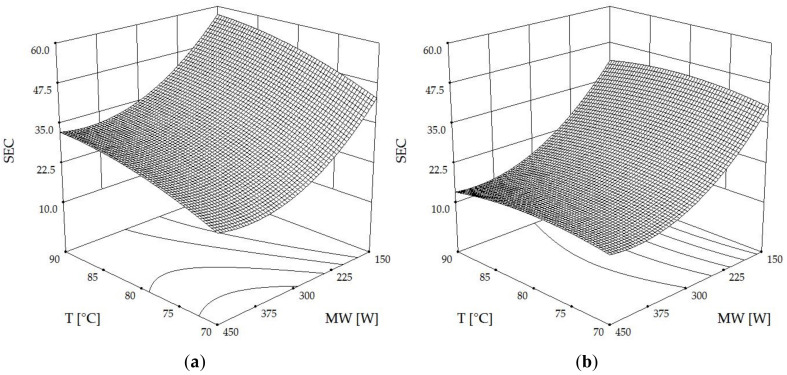

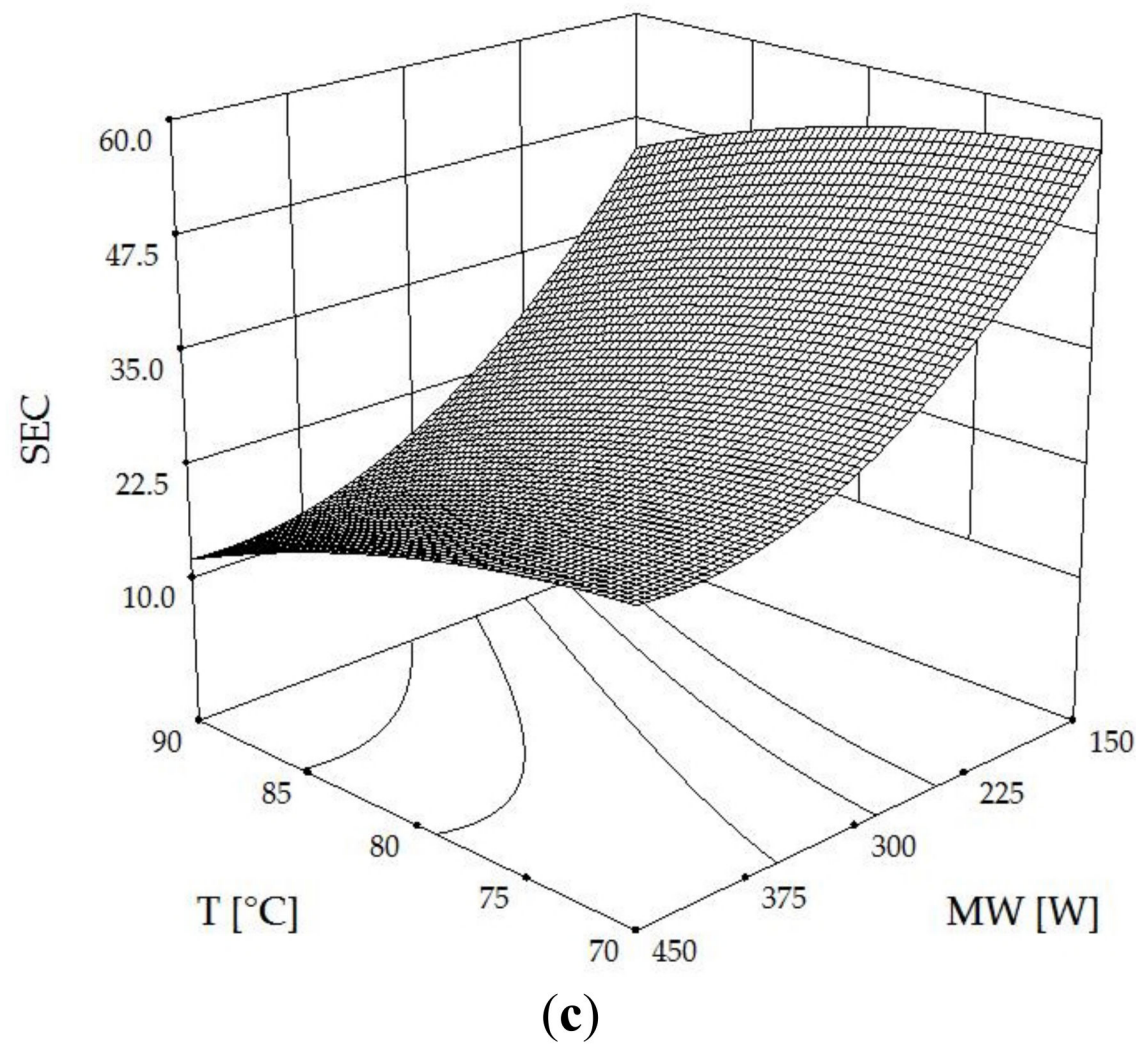
Three-dimensional contour plots of SEC (MJ·(kg·h^−1^)^−1^) affected by hot air temperature (T) and microwave power (MW) at rotational speeds (RDS) of (**a**) 0.07 Hz, (**b**) 0.13 Hz, and (**c**) 0.20 Hz.

As depicted in Figure 5a–c, the relationship between drying air temperature, microwave power, and rotational speed plays a pivotal role in determining the specific energy consumption (SEC). SEC, which measures the total energy utilized per kilogram of evaporated water to reduce moisture content to 10% (wb), was found to be lowest at the highest temperature and microwave power across all rotational speeds. Notably, at higher rotation speeds (Figure 5c), microwave intensity exerted a more pronounced influence on SEC compared to temperature. This can be attributed to the fact that higher microwave energy leads to rapid drying and shorter drying times, thereby reducing SEC. This aligns with the findings in [7,34], where the effective use of microwave power significantly enhances drying efficiency and moisture evaporation, resulting in reduced drying times. Furthermore, the rotational disc speed (RDS) was observed to increase SEC at faster speeds. This could be explained by the theory discussed in [12], which indicates that higher rotation speeds in microwave-rotary drying systems improve moisture diffusivity and drying efficiency. However, this increase in efficiency comes at the cost of higher energy consumption due to the energy required to maintain higher rotational speeds, as suggested in the findings of [11]. Therefore, while higher microwave intensities and temperatures contribute to lower SEC by facilitating faster drying, the increase in rotational speed, although beneficial for uniform drying and moisture diffusivity, tends to raise SEC, reflecting the energy trade-offs in the drying process.

The data presented in Table 3 and the various contour and diagnostic plots indicated that the quadratic polynomial model was a suitable tool for navigating design space and optimization. However, removing any insignificant terms also enhanced model accuracy.

### 3.3. Multiple Response Optimization

Previous studies revealed that the effective diffusivity coefficient (D_e_), and specific energy consumption (SEC) were influenced by hot air temperature (T), microwave power (MW), and rotational speed (RDS) in different ways. The best combination of these variables was explored to optimize the process. The goal of this study was to determine the optimal values for all variables by maximizing D_e_ and minimizing SEC using a desirability function. 

**Figure 6:**
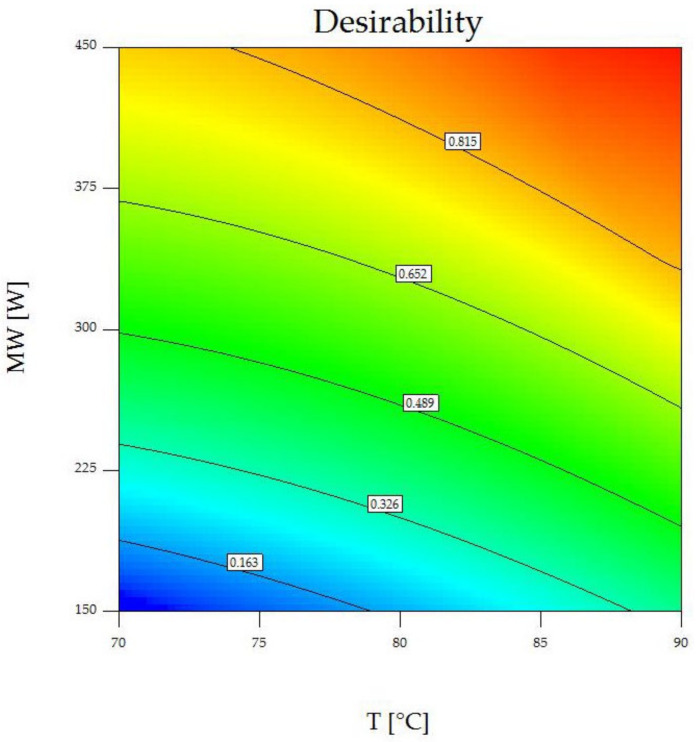
Contour plot of the desirability function at a fixed rotational disc speed of 0.20 Hz. Color represents the value, ranging from minimum value of 0 (blue) to maximum value of 1 (red).

Figure 6 shows a contour plot of the desirability function at a rotational disc speed of 0.20 Hz, presenting the best area for producing desirable results. The combination of a hot air temperature of 90 °C, a microwave power of 450 W (initial intensity of 2.25 W·g^−1^), and a rotational speed of 0.20 Hz resulted in the highest desirability of 0.978, making it the globally optimal method for convective hot-air drying combined with microwave heating to produce quick-cooking red beans.

### 3.4. Drying Performance and Quality Attributes Affected by Stepwise Microwave Heating

The drying processes operated at high microwave energy were suitable for producing quick-cooking red beans (QCRB) with respect to energy consumption, but quality attributes may be impacted by overheating or charring. Therefore, changing the microwave power during the drying process could contribute to avoid this problem.

The complete semi-empirical Page drying model (Equation (2)) was used to estimate drying times for each stage, as shown in Table 4. The drying period was divided into two steps using 70% of the initial moisture content [22,23] as an intermediate point for changing microwave intensity. After reaching such a point, the sample was further dried under 90 °C combined with stepped-up/stepped-down microwave heating until its moisture content was reduced to approximately 10% (wb). Two modes of stepwise drying were studied, as shown in Table 4.

Table 5 shows the water evaporation rate (W_evap_), energy consumption, water evaporation rate per unit of energy used (SMER), and specific energy consumption per unit of evaporated water (SEC) in different drying conditions.

The water evaporation rate per unit of time (W_evap_), serves as a performance indicator for a drying efficiency to evaporate moisture in a certain period. The analysis of the data presented in Table 5 reveals varying rates of water evaporation from the sample material under different drying conditions, ranging between 0.095 ± 0.001 kg·h^−1^ and 0.316 ± 0.012 kg·h^−1^. Notably, drying conditions utilizing a constant microwave power of 450 W exhibited the highest W_evap_, while those involving a step-down approach with microwave power reduced from 450 W to 150 W, or from 300 W to 150 W, demonstrated the lowest W_evap_ values. These experimental findings can be attributed to the higher thermal energy generation through a higher power (450 W), resulting from an intensified electromagnetic energy. Conversely, after the initial drying phase under conditions at 450 W and 300 W in the step-down mode, the power was adjusted to 150 W for the majority of the drying period, specifically accounting for approximately 88% and 83% of the total duration, respectively. This drying mechanism aligned with earlier research studies conducted by [23,35,36,37].

The process of drying is known to require a substantial amount of energy. In this study, an alternative approach was employed, wherein the hot-air drying process was combined with stepwise microwave heating. The primary energy consumption in this combined method resulted from the heating source, which employed hot-air and microwave heating systems. The investigation revealed that the total energy consumption varied under different drying conditions. Specifically, when stable drying conditions were maintained with a fixed microwave power of 450 W, the total energy consumption ranged from 0.87 ± 0.00 kWh to 1.02 ± 0.01 kWh, equivalent to 3.13 ± 0.00–3.67 ± 0.05 MJ. Notably, the lowest energy consumption was observed in instances with a minimum drying time of merely 1195 s, approximately 20 min.

The evaluation of energy consumption performance in the drying system commonly involves the utilization of specific moisture extraction rate (SMER) and specific energy consumption (SEC) values. Higher SMER values and lower SEC values are indicative of better energy efficiency in the drying process. Upon examining the data presented in Table 5, it was found that the SMER values ranged from 0.095 ± 0.001 to 0.363 ± 0.013 kg·h^−1^·(kWh^−1^), while the SEC values varied from 9.91 ± 0.36 to 37.74 ± 0.49 MJ·(kg·h^−1^)^−1^. Taking energy efficiency into account, the most suitable drying condition was identified to be when a fixed microwave power of 450 W was utilized. This conclusion was supported by the fact that such conditions resulted in higher microwave power consumption, generating an abundance of internal heat, which, in turn, led to an accelerated drying rate during shorter drying periods [23,35,36].

The analysis of drying conditions for efficiently preparing quick-cooking red beans took into consideration not only energy efficiency but also the physical quality and rehydration attributes. Consumer acceptance is greatly influenced by the latter attributes. Therefore, the selection of appropriate drying conditions necessitated a thorough evaluation of the final product quality. Table 6 and Table 7 present the quality assessment of red beans under various drying conditions and the subsequent evaluation of rehydration, appearance, and texture after rehydrating quick-cooking grains using different drying methods. The tables clearly indicate that different drying conditions yielded distinct qualities in terms of the dried red beans and their rehydration properties.

The results in Table 6 demonstrated that the final moisture content of all samples fell within the range of 10.24–11.92% wb, which closely aligned with the predicted value and the moisture content of the raw material (11.68%). The moisture content of the quick-cooking red beans, dried using the freeze-drying method, was 3.53%, consistent with those obtained elsewhere [38,39]. Furthermore, Table 6 presents the water activity (a_w_) values of the samples dried by various methods, ranging from 0.4149 to 0.5541, indicating safe storage conditions (<0.6). The a_w_ values of the raw red beans and the quick-cooking samples dried using freeze-drying were 0.3940 and 0.2419, respectively.

The physical properties of dried products are important in determining consumer acceptance. Table 6 presents essential parameters such as breakage percentage (%Break), shrinkage percentage (%Shrink), color values, and color difference value (ΔE).

The breakage percentage ranged from 32.63 ± 3.22% to 45.74 ± 3.57% when using a fixed microwave power of 450 W. A comparative analysis of stepwise drying conditions revealed statistically insignificant differences in the percentage of breakage observed in QCRB (*p* > 0.05). This observed similarity in %breakage value can be attributed to the use of 450 W throughout the drying process. During the initial drying phase, a rapid reduction in moisture content occurred within the material. However, in the subsequent stages of drying, even with the sustained high microwave power, the sample moisture remained low. Consequently, this phenomenon might induce puffing mechanisms until the grain breaks [40,41,42]. Specifically, the %breakage value of QCRB samples produced using freeze-drying was only 15.48 ± 3.08%.

The shrinkage of food material during the drying process is a critical factor that significantly influences the quality of dried products, particularly affecting attributes such as texture and taste. Various factors, including the specific drying conditions, exert a notable impact on this shrinkage phenomenon [43,44]. According to Table 6, it became evident that the shrinkage percentage varied when subjected to different microwave power levels during the drying process. Specifically, as the step-up microwave heating increased from 150 W to 300 W, the shrinkage percentage was 43.79 ± 2.86%, whereas the step-down mode decreasing from 450 W to 300 W resulted in %shrinkage values of 44.17 ± 3.04%. On the other hand, the minimum %shrinkage of 22.80 ± 2.17% was obtained from using 450 W for the first stage and 150 W for the later stage. This low value was slightly higher than that of the sample prepared using a freeze-drying process (16.78 ± 0.48%), which is considered as an ideal process to produce dried products with minimal shrinkage.

The majority of consumers tends to favor processed products that maintain their natural coloration without appearing excessively pale or bleached. In this study, a color change value (ΔE) was employed as one of the parameters to evaluate the efficiency of the drying process. As indicated in Table 6, various drying conditions led to changes in lightness (L*), redness (a*), and yellowness (b*), leading to varied ΔE values. The L*, a*, and b* values of the dried red bean samples were compared to the inherent color of the raw red bean material. Table 6 reveals that the ΔE values fell within the range of 1.35 ± 0.07 to 7.76 ± 0.56, with the step-down mode reducing microwave power from 300 W to 150 W resulting in the lowest ΔE value, while the other drying modes gave a ΔE higher than 3 [45]. The highest ΔE values were observed when using a high microwave power of 450 W and using the step-up mode from 150 W to 450 W. This significant color change can be attributed to the use of a fixed power during the drying process or the application of high microwave energy during the final drying stages, leading to a reduction in the material weight due to water evaporation. While the power output remained constant, the microwave energy density intensified, resulting in excessive heat within the material, leading to burning and eventual color change [46].

Based on the data presented in Table 6, it can be concluded that employing a constant microwave power of 450 W throughout the drying process proved to be energy-efficient, but providing an alteration of the physical characteristics of the dried product, when compared to those of raw materials or samples produced using freeze-drying, which is regarded as the superior drying approach.

Rehydration and textural properties are important properties of instant or quick-cooking products. Table 7 shows the rehydration time (RT), rehydration ratio (RR), and textural properties of rehydrated samples, prepared using various methods.

From Table 7, it was found that the RT value of samples prepared using different drying processes was not significantly different. The maximum RT of 6 min was obtained when drying with a fixed microwave power output of 450 W, possibly due to the case-hardening of the grain surface which resulted from an imbalance between moisture migration from the interior to the surface and moisture diffusion from the surface to the ambient. The shortest RT was obtained when the product was prepared using freeze-drying, which was due to higher porosity, resulting in faster water absorption during the rehydration process. Considering the RR values from Table 7, it can be seen that the RR value was consistent with the rehydration time. The lowest RR value (1.08 ± 0.06) was obtained using the fixed microwave power output of 450 W. A slower water absorption, hindered by the case-hardening of the grain surface, was also responsible for this low RR value [47].

The textural properties of the rehydrated quick-cooking red beans were compared with those of the samples prepared using the traditional cooking method and freeze-drying. From Table 7, it was found that the texture values of red beans that had been dried using the proposed method had different values when the drying conditions were different. Here, the hardness values were in the range of 43,449 ± 2469–50,256 ± 1705 g, which was comparable to those of traditionally cooked red beans (51,770 ± 1643 g), while samples dried using freeze drying had a slightly lower value (35,244 ± 821 g). The stickiness value (4.71 ± 1.41–30.13 ± 12.33) was obviously lower compared to that observed after freeze drying. From Table 7, it can be concluded that all drying conditions, except those using a fixed microwave intensity of 450 W throughout the drying period, produced textural properties of rehydrated quick-cooking red beans which were comparatively close to those of the traditionally cooked samples.

### 3.5. Optimal Drying Process Obtained through Synthetic Evaluation Analysis

The appropriate drying approach was evaluated using the synthetic evaluation index (S), using the criteria based on energy consumption and consumer preference aspects. Table 8 shows the S values obtained from various drying circumstances.

The data presented in Table 8 offer a synthetic evaluation index, taking into account the energy consumption criterion together with the remaining consumer preferences under various drying conditions. This table reveals that S values range between approximately 0.86 ± 0.00 and 1.97 ± 0.05. Notably, the maximum S value was observed under a step-down drying scenario with using a microwave power of 450 W for the first stage and 300 W for the second stage, implying that the optimal drying scenario was chiefly dictated by the predominant energy consumption, followed by the remaining acceptable product qualities. Consequently, the optimal conditions for drying can be characterized as a hot-air drying process at 90 °C, in combination with microwave heating starting from 450 W for approximately 8 min, then 300 W for the later drying duration, which extended to approximately 28 min, and a rotational disc speed of 0.20 Hz.

## 4. Conclusions

This research introduced a combination of hot-air and stepwise microwave drying as an effective, energy-efficient method for producing quick-cooking red beans, while preserving their rehydration and textural attributes. The synergistic drying approach facilitated a shorter drying time, a higher effective diffusivity coefficient, and lower specific energy consumption compared to hot-air drying alone. The optimal drying process was determined via response surface methodology, yielding a maximized effective diffusivity coefficient and minimized drying time and specific energy consumption. Crucial findings comprised the description of optimal drying conditions favoring reduced energy consumption, encompassing a hot air temperature of 90 °C, a microwave power of 450 W, and a rotational disc speed of 0.20 Hz. Nevertheless, elevated microwave intensity precipitated a degradation in quality owing to the excessive heat generation, causing burns and alterations in physical properties, especially color. Additionally, this research underscored the effectiveness of stepwise microwave heating as a preventive measure against overheating, consequentially averting burns and quality degradation during the drying process. The synthetic evaluation index revealed that stepwise drying conditions—wherein microwave power was diminished from 450 W to 150 W or from 300 W to 150 W—emerged as a balanced approach, striking a fine line between minimal energy consumption and the quality preservation of the end product compared to traditionally cooked red beans and those produced through the superior freeze-drying process. These findings have pivotal implications for enhancing drying performance and energy efficiency across other food products. Additionally, they offer a novel standpoint on quick-cooking bean preparation methods, aligning with the abrupt pace of modern lifestyles without trading off the beans’ quality attributes.

## Figures and Tables

**Table 1:** Stepwise modes for microwave heating with different optimized constant powers (W).

No.	Stepwise Mode	Stage 1	Stage 2
1	constant	MW	MW
2	step up	150	300
3	step up	150	450
4	step up	300	450
5	step down	450	150
6	step down	450	300
7	step down	300	150

**Table 2:** Drying conditions and their responses.

Drying Conditions			Responses	
T	MW	RDS	D_e_	SEC
70	150	0.07	0.64	38.50
70	150	0.13	0.61	27.85
70	150	0.20	0.53	68.06
70	300	0.07	1.25	29.94
70	300	0.13	1.68	22.90
70	300	0.20	1.34	25.74
70	450	0.07	1.79	20.07
70	450	0.13	3.18	17.99
70	450	0.20	2.80	24.89
80	150	0.07	0.86	56.59
80	150	0.13	0.84	42.44
80	150	0.20	0.75	61.10
80	300	0.07	1.50	32.03
80	300	0.13	1.46	19.90
80	300	0.20	1.58	22.83
80	450	0.07	3.04	25.91
80	450	0.13	2.64	19.94
80	450	0.20	2.33	18.03
90	150	0.07	0.89	61.81
90	150	0.13	0.89	40.16
90	150	0.20	1.31	36.78
90	300	0.07	1.56	35.46
90	300	0.13	1.74	21.11
90	300	0.20	2.82	19.65
90	450	0.07	2.21	26.37
90	450	0.13	2.99	16.58
90	450	0.20	2.87	18.08

T—temperature (°C); MW—microwave power (W); RDS—rotational disc speed (Hz); D_e_—effective diffusivity (×10^−9^ m^2^·s^−1^); SEC—specific energy consumption (MJ·(kg·h^−1^)^−1^).

**Table 3:** ANOVA results of fitted models for all responses.

Source	Estimate Coefficients	
	D_e_	SEC
Model (*p* value)	<0.0001 ^c^	<0.0001 ^c^
Intercept		
(a_0_)	1.70	20.93
Linear terms		
(a_1_) X_1_	0.20 ^a^	−0.17 ^ns^
(a_2_) X_2_	0.92 ^c^	−13.68 ^c^
(a_3_) X_3_	0.14 ^ns^	−1.75 ^ns^
Interaction terms		
(a_12_) X_1_X_2_	−0.084 ^ns^	−0.52 ^ns^
(a_13_) X_1_X_3_	0.12 ^ns^	−6.65 ^b^
(a_23_) X_2_X_3_	0.057 ^ns^	−1.86 ^ns^
Quadratic terms		
(a_11_) $X_{1}^{2}$	0.061 ^ns^	−2.53 ^ns^
(a_22_) $X_{2}^{2}$	0.073 ^ns^	9.00 ^b^
(a_33_) $X_{3}^{2}$	−0.12 ^ns^	9.31 ^b^
F value		
Model	13.37	12.16
X_1_	4.96	4.39 × 10^−6^
X_2_	110.05	73.44
X_3_	2.70	1.21
X_1_X_2_	0.61	0.072
X_1_X_3_	1.23	11.50
X_2_X_3_	0.28	0.76
$X_{1}^{2}$	0.16	0.85
$X_{2}^{2}$	0.23	10.67
$X_{3}^{2}$	0.64	10.94
R^2^	0.8762	0.8682
Adj.R^2^	0.8107	0.7984
Predicted R^2^	0.6656	0.6004
Adeq. Precision	11.41	11.12
C.V. (%)	21.78	21.21
Std. Dev.	0.37	6.68

^a^, ^b^, and ^c^ represent significant difference at *p* < 0.05, *p* < 0.005, and *p* < 0.001, respectively. ‘ns’ stands for ‘not significant’.

**Table 4:** Estimated stepwise drying times at a fixed temperature of 90 °C and a rotation speed of 0.20 Hz.

No.	Stage 1		Stage 2	
	MW	Time	MW	Time
1	450	1195	-	-
2	150	1288	300	1663
3	150	1288	450	1054
4	300	671	450	1054
5	450	447	150	3281
6	450	447	300	1663
7	300	671	150	3281

MW stands for microwave power (W). Time was expressed in seconds. ‘-‘ symbol means no change in microwave power.

**Table 5:** Energy consumption of all drying scenarios.

MW		W_evap_	Energy Consumed		SMER	SEC
Stage 1	Stage 2		(kWh)	(MJ)		
450	-	0.316 ± 0.012 ^a^	0.87 ± 0.00 ^d^	3.13 ± 0.00 ^d^	0.363 ± 0.013 ^a^	9.91 ± 0.36 ^g^
150	300	0.127 ± 0.001 ^e^	1.02 ± 0.01 ^a^	3.67 ± 0.05 ^a^	0.124 ± 0.002 ^e^	28.92 ± 0.58 ^c^
150	450	0.159 ± 0.002 ^d^	0.97 ± 0.00 ^bc^	3.49 ± 0.00 ^bc^	0.163 ± 0.002 ^d^	22.01 ± 0.22 ^d^
300	450	0.216 ± 0.004 ^b^	0.96 ± 0.00 ^c^	3.46 ± 0.00 ^c^	0.225 ± 0.004 ^b^	15.98 ± 0.30 ^f^
450	150	0.101 ± 0.001 ^f^	1.01 ± 0.04 ^a^	3.64 ± 0.15 ^a^	0.100 ± 0.006 ^f^	36.15 ± 2.01 ^b^
450	300	0.183 ± 0.001 ^c^	0.99 ± 0.01 ^ab^	3.56 ± 0.05 ^abc^	0.184 ± 0.002 ^c^	19.53 ± 0.22 ^e^
300	150	0.095 ± 0.001 ^f^	1.00 ± 0.00 ^a^	3.60 ± 0.00 ^ab^	0.095 ± 0.001 ^f^	37.74 ± 0.49 ^a^

MW stands for microwave power (W). W_evap_ is the rate of water evaporation (kg·h^−1^); SMER and SEC stand for specific moisture evaporation rate (kg·h^−1^·(kWh^−1^)) and specific energy consumption (MJ·(kg·h^−1^)^−1^). Superscripts represent a significant difference of the means in the same column (*p* < 0.05). ‘-‘ symbol means no change in microwave power.

**Table 6:** Quality attributes of quick-cooking red beans at different drying conditions.

Stepwise Mode		MC (% w.b.)	a_w_	%Break	%Shrinkage	Color			
Stage 1	Stage 2					L*	a*	b*	ΔE
450	-	10.74 ± 0.93	0.4167 ± 0.002	45.74 ± 3.57 ^a^	32.76 ± 2.38 ^d^	69.48 ± 0.78 ^a^	1.44 ± 0.07 ^d^	10.33 ± 0.05 ^d^	7.76 ± 0.56 ^a^
150	300	10.24 ± 0.01	0.4149 ± 0.001	33.17 ± 0.94 ^b^	43.79 ± 2.86 ^a^	65.75 ± 1.68 ^d^	1.46 ± 0.13 ^d^	9.27 ± 0.25 ^g^	5.08 ± 0.85 ^d^
150	450	11.78 ± 0.07	0.5233 ± 0.004	37.04 ± 1.71 ^b^	37.92 ± 1.82 ^bc^	69.45 ± 0.22 ^a^	1.69 ± 0.02 ^c^	10.30 ± 0.03 ^d^	7.67 ± 0.30 ^a^
300	450	11.92 ± 0.87	0.5476 ± 0.008	35.57 ± 2.63 ^b^	36.49 ± 1.16 ^c^	68.23 ± 0.79 ^ab^	1.46 ± 0.05 ^d^	9.91 ± 0.12 ^e^	6.73 ± 0.48 ^b^
450	150	11.55 ± 0.48	0.4752 ± 0.003	33.80 ± 1.08 ^b^	22.80 ± 2.17 ^e^	66.84 ± 0.31 ^cd^	1.54 ± 0.01 ^d^	9.54 ± 0.05 ^f^	5.64 ± 0.16 ^cd^
450	300	10.90 ± 0.44	0.4524 ± 0.002	35.31 ± 1.85 ^b^	44.17 ± 3.04 ^a^	67.55 ± 0.70 ^bc^	1.73 ± 0.08 ^c^	9.93 ± 0.06 ^e^	6.03 ± 0.07 ^c^
300	150	11.53 ± 0.47	0.5541 ± 0.007	32.63 ± 3.22 ^b^	41.06 ± 0.42 ^ab^	62.13 ± 0.37 ^e^	2.42 ± 0.01 ^b^	11.43 ± 0.08 ^c^	1.35 ± 0.07 ^e^
Raw		11.68 ± 0.07	0.3940 ± 0.003	-	-	62.24 ± 0.26 ^e^	3.60 ± 0.06 ^a^	12.08 ± 0.03 ^b^	-
Freeze-dried		3.53 ± 0.11	0.2419 ± 0.006	15.48 ± 3.08 ^c^	16.78 ± 0.48 ^f^	62.26 ± 0.20 ^e^	3.62 ± 0.00 ^a^	13.08 ± 0.01 ^a^	1.00 ± 0.02 ^e^

Superscripts represent a significant difference of the means in the same column (*p* < 0.05). ‘-‘ symbol means no change in microwave power.

**Table 7:** Rehydration and textural properties of quick-cooking red beans prepared using different drying conditions.

Stepwise Mode		Rehydration Time (min)	Rehydration Ratio	Textural Properties	
Stage 1	Stage 2			Hardness (g)	Stickiness (g·s)
450	-	6.00 ± 0.00 ^a^	1.08 ± 0.06 ^c^	43,449 ± 2469 ^c^	30.13 ± 12.33 ^b^
150	300	5.67 ± 0.58 ^ab^	1.34 ± 0.02 ^b^	49,240 ± 1044 ^ab^	7.84 ± 1.23 ^c^
150	450	5.33 ± 0.58 ^abc^	1.36 ± 0.17 ^ab^	49,333 ± 2139 ^ab^	8.13 ± 2.10 ^c^
300	450	4.67 ± 0.58 ^cde^	1.34 ± 0.02 ^b^	49,342 ± 2866 ^ab^	4.71 ± 1.41 ^c^
450	150	5.00 ± 0.00 ^bcd^	1.41 ± 0.01 ^ab^	50,256 ± 1705 ^a^	8.06 ± 3.29 ^c^
450	300	4.33 ± 0.58 ^de^	1.46 ± 0.07 ^ab^	46,257 ± 1447 ^bc^	4.82 ± 2.42 ^c^
300	150	5.00 ± 0.00 ^bcd^	1.35 ± 0.09 ^ab^	50,134 ± 2546 ^a^	9.23 ± 2.18 ^c^
Conventionally cooked		-	-	51,770 ± 1643 ^a^	17.64 ± 9.65 ^bc^
Freeze-dried		4.00 ± 0.00 ^e^	1.49 ± 0.01 ^a^	35,244 ± 821 ^d^	194.25 ± 15.65 ^a^

Superscripts represent a significant difference of the means in the same column (*p* < 0.05). ‘-‘ symbol means no change in microwave power.

**Table 8:** Synthetic evaluation index obtained from all drying scenarios.

MW		S
Stage 1	Stage 2	
450	-	0.86 ± 0.00 ^f^
150	300	1.24 ± 0.20 ^e^
150	450	1.41 ± 0.08 ^de^
300	450	1.89 ± 0.05 ^ab^
450	150	1.71 ± 0.12 ^bc^
450	300	1.97 ± 0.05 ^a^
300	150	1.49 ± 0.22 ^cd^

Superscripts represent a significant difference of the means in the same column (*p* < 0.05). ‘-‘ symbol means no change in microwave power.

## Data Availability

The original contributions presented in the study are included in the article, further inquiries can be directed to the corresponding author.
